# Protection of human research participants through routine inspection of ongoing HIV prevention and treatment clinical trials: experiences from Zimbabwe

**DOI:** 10.1186/1742-4690-9-S2-P228

**Published:** 2012-09-13

**Authors:** RB Zinyama-Gutsire, R Musesengwa, S Ruzario, R Gunda

**Affiliations:** 1 Medical Research Council of Zimbabwe, Harare, Zimbabwe

## Background

The Medical Research Council of Zimbabwe (MRCZ) is the National Ethics Committee in Zimbabwe. MRCZ was established in 1974 under the Research Act of 1959 and Government Notice No. 225 of 1974 to promote and co-ordinate health research in Zimbabwe. The main mandate of MRCZ is the scientific review, ethics approval and oversight of all ongoing medical research studies in Zimbabwe inspection of all on going research studies involving humans using an established Human Protection System. Routine inspections and ‘for cause’ inspections are carried out to ensure utmost protection of research participants and to ensure protocol adherence and ICH-GCP compliance. HIV prevention and HIV drug treatment clinical trials receive more detailed attention during inspections as they are delicate and complicated.

## Methods

During the inspection process the following aspects are checked on and thoroughly reviewed: initial and continuing MRCZ approval letters, protocol version at study site, National Drug Regulatory Body approval in case of drug clinical trials, valid continuing review approvals, staff qualifications, staff registration with professional bodies and relevant experience, evidence of research ethics and GCP training, data storage, signed and dated participant consent forms, external monitors reports and recommendations, approved study data collection tools, procedure rooms and interviews study participants. MRCZ adherence to Local and International Research Ethics Guidelines and offers research ethics and GCP training courses to local researchers.

## Results

A recent development has been proactive requests from researchers for research ethics and GCP training, an indication of the increased awareness amongst researchers in Zimbabwe of the need for this training.

## Conclusion

There has been significant improvement in protection of research participants, protocol adherence and ICH-GCP compliance by researchers as a result of the increased inspection of all ongoing research studies in Zimbabwe. More and more researchers are now aware of international research.

